# Urethrovesical calcified foreign body: Case report and literature review

**DOI:** 10.1016/j.ijscr.2024.110794

**Published:** 2025-01-07

**Authors:** Salim Ouskri, Youssef Zaoui, Ahmed IBRAHIMI, Imad Boualaoui, Hachem El Sayegh, Yassine Nouini

**Affiliations:** IBN SINA University Hospital, Morocco

## Abstract

**Introduction:**

Calcified bladder foreign bodies are a rare yet clinically significant entity, often introduced voluntarily or in psychiatric contexts. Their diagnosis is frequently delayed due to nonspecific symptoms and the concealed circumstances of their introduction.

**Case presentation:**

We report the case of a 37-year-old man, treated for schizophrenia, presenting with chronic urinary symptoms. Imaging revealed bladder calculi, later identified as calcified deposits surrounding a plastic bracelet introduced by the patient.

**Discussion:**

The case highlights the diagnostic challenges and therapeutic approaches, including surgical removal and psychiatric care to prevent recurrence. A multidisciplinary approach is recommended for optimal outcomes.

**Conclusion:**

Calcified bladder foreign bodies are a rare but clinically relevant phenomenon, often associated with psychiatric disorders. Their management requires accurate diagnosis based on modern imaging techniques and tailored surgical intervention. Postoperative psychiatric care is essential to prevent high-risk behaviors and improve long-term outcomes.

## Highlights

• Rare Occurrence: Urethrovesical foreign bodies are uncommon but pose significant diagnostic and therapeutic challenges, especially when calcified over time.
• Psychiatric Associations: Many cases are linked to psychiatric conditions, including schizophrenia and depression, necessitating integrated mental health care.
• Diagnostic Tools: Imaging techniques like ultrasound, X-ray, CT, and cystoscopy are essential for identifying and differentiating calcified foreign bodies from bladder stones.
• Surgical Management: Open surgery is required for large or encrusted foreign bodies, while endoscopic methods are preferred for simpler cases.
• Prevention of Recurrence: Multidisciplinary management, including psychiatric evaluation and follow-up, is crucial to address underlying causes and prevent recurrent behaviors.

## 1 Introduction

Urethrovesical foreign bodies, although rare, present a diagnostic and therapeutic challenge in urology. They are generally introduced voluntarily in erotic or psychiatric contexts but can also result from accidents or medical interventions. The calcification of these objects, caused by prolonged reactions of the bladder mucosa, further complicates their early identification. These cases often require interdisciplinary collaboration between urologists and psychiatrists for effective management [[1], [2], [3]]. The presence of these foreign bodies can lead to various complications, including chronic infections, hematuria, and, in severe cases, vesicocutaneous fistulas [4]. Understanding the associations between psychiatric disorders and foreign body insertion is crucial, as many cases are linked to mental health conditions such as schizophrenia and depression [2, 5]. These observations underline the importance of integrated psychiatric management to prevent recurrent behaviors [1,3].

## 2 Case presentation

We present the case of a 37-year-old male who consulted our outpatient clinic with persistent chronic dysuria and a burning sensation during micturition. Notably, the patient had a long-standing history of schizophrenia managed with treatment for seven years, and he denied any previous urethral manipulations.

**Fig. 1:**
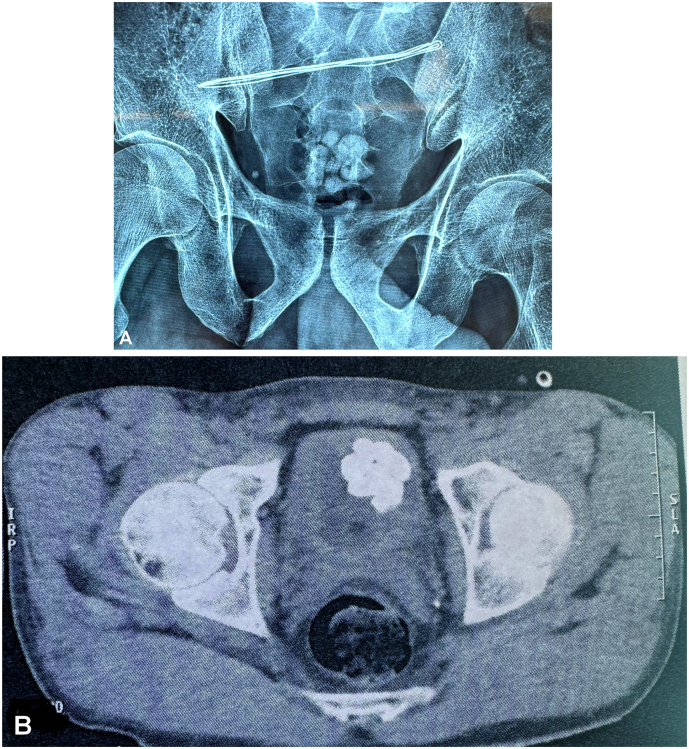
A - Image showing calcific shadows projected over the bladder area. B - Image showing multiple calculi in the bladder.

Clinical examination was unremarkable, but imaging results painted a puzzling picture. A pelvic ultrasound revealed multiple bladder calculi without renal abnormalities, which was further confirmed by a plain abdominal X-ray (AUSP) showing radiopaque stones. An abdominopelvic CT scan corroborated these findings, identifying what appeared to be multiple large bladder stones, with no evidence of renal lithiasis (Fig. 1).

**Fig. 2:**
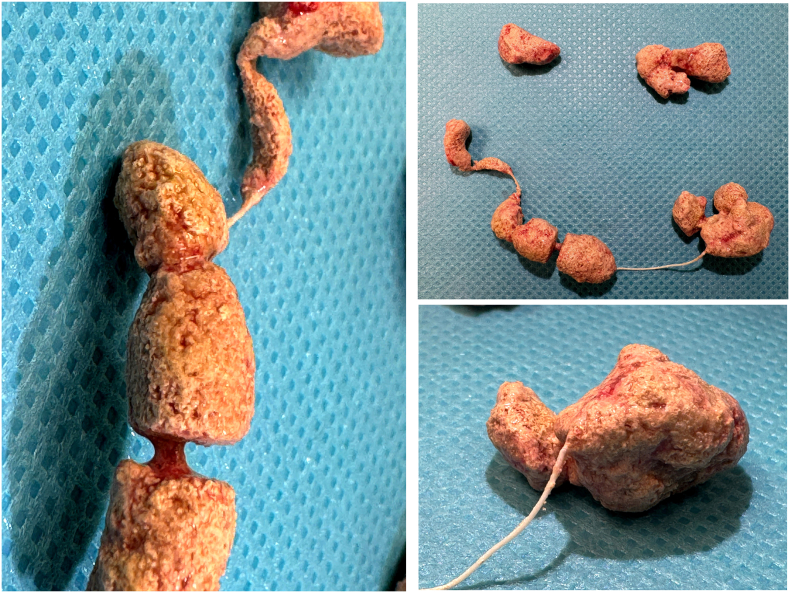
Images of the calcified bracelet after extraction.

Given the imaging results and clinical symptoms, the patient was scheduled for a suprapubic cystotomy. However, the intraoperative findings revealed a surprising twist: the suspected “stones” were, in fact, a foreign body. A plastic bead bracelet, introduced into the bladder by the patient himself years earlier, had undergone significant calcification over time, mimicking bladder stones (Fig. 2).

## 3 Discussion

The presence of calcified foreign bodies in the bladder has been documented in several similar cases. Walsh and Moustafa reported that these objects are often introduced voluntarily for masturbatory purposes or in psychiatric contexts [1]. The case presented highlights the diagnostic complexity associated with delayed medical care due to embarrassment or underlying psychological disorders.

The diagnosis relies on a combination of imaging modalities, including ultrasound, radiography, and CT, which help differentiate simple calcifications from foreign bodies. Cystoscopy remains the gold standard for confirming the presence of and strategizing the removal of the object. In this case, extensive calcification made the identification of the initial object challenging until surgical exploration.

Therapeutic management is primarily surgical, although endoscopic approaches are preferred in simpler cases. However, open surgery becomes necessary when foreign bodies are large or heavily encrusted.

Complications associated with bladder foreign bodies include chronic urinary infections, hematuria, and, in advanced cases, vesicocutaneous fistulas. Early intervention is critical to prevent these severe outcomes. Moreover, studies have highlighted the role of minimally invasive techniques in extracting foreign bodies, providing an effective alternative to open surgery in less complex cases [2,3].

A review of the literature reveals that these cases are frequently associated with underlying psychiatric disorders. For example, Bosquet Sanz et al. emphasized that most patients suffer from schizophrenia, depression, or personality disorders [2]. These observations underline the importance of integrated psychiatric management to prevent recurrent behaviors. A multidisciplinary approach, including routine psychiatric consultation after intervention, is therefore recommended to optimize long-term outcomes [1,4].

Finally, it is noteworthy that calcified foreign bodies can sometimes remain undetected for years, as symptoms are often nonspecific. This highlights the importance of thorough history-taking and imaging in the presence of atypical urinary symptoms, even when patients deny any known manipulation.

## 4 Conclusion

Calcified bladder foreign bodies are a rare but clinically relevant phenomenon, often associated with psychiatric disorders. Their management requires accurate diagnosis based on modern imaging techniques and tailored surgical intervention. Postoperative psychiatric care is essential to prevent high-risk behaviors and improve long-term outcomes.

## Methods

This the work has been reported in line with the SCARE criteria.

## CRediT authorship contribution statement

Salim Ouskri **Urology Resident: FIRST author has contributed in the writing and correction the case report.**

Youssef Zaoui **Urology Resident: has contributed in the correction the case report.**

Imad Boualaoui **Urology assistant Professor/has contributed in the correction the case report.**

Ahmed IBRAHIMI **Urology assistant Professor: SECOND author has contributed in the writing and correction the case report.**

Hachem El Sayegh **Urology Professor: has contributed in the correction the case report.**

Yassine Nouini **Urology Professor: has contributed in the correction the case report.**

## Consent

Patient consent is obtained

## Ethical approval

Ethical approval is obtained from the ethical comity of the hospital.

## Guarantor

Salim Ouskri.

## Sources of funding

no source of funding.

## Declaration of competing interest

I declare no conflict of interest.
